# Comparative Analysis of Orthosteric and Allosteric GLP-1R Agonists’ Effects on Insulin Secretion from Healthy, Diabetic, and Recovered INS-1E Pancreatic Beta Cells

**DOI:** 10.3390/ijms25126331

**Published:** 2024-06-07

**Authors:** Joshua Reed, Victoria Higginbotham, Stephen Bain, Venkateswarlu Kanamarlapudi

**Affiliations:** Institute of Life Science, Medical School, Swansea University, Singleton Park, Swansea SA2 8PP, UK; joshjreed@hotmail.co.uk (J.R.); v.higginbotham@swansea.ac.uk (V.H.); s.c.bain@swansea.ac.uk (S.B.)

**Keywords:** diabetes, INS-1E cells, glucose, GLP-1R, allosteric agonists, orthosteric agonists, GSIS, GSICP, insulin

## Abstract

Despite the availability of different treatments for type 2 diabetes (T2D), post-diagnosis complications remain prevalent; therefore, more effective treatments are desired. Glucagon-like peptide (GLP)-1-based drugs are currently used for T2D treatment. They act as orthosteric agonists for the GLP-1 receptor (GLP-1R). In this study, we analyzed in vitro how the GLP-1R orthosteric and allosteric agonists augment glucose-stimulated insulin secretion (GSIS) and intracellular cAMP production (GSICP) in INS-1E pancreatic beta cells under healthy, diabetic, and recovered states. The findings from this study suggest that allosteric agonists have a longer duration of action than orthosteric agonists. They also suggest that the GLP-1R agonists do not deplete intracellular insulin, indicating they can be a sustainable and safe treatment option for T2D. Importantly, this study demonstrates that the GLP-1R agonists variably augment GSIS through GSICP in healthy, diabetic, and recovered INS-1E cells. Furthermore, we find that INS-1E cells respond differentially to the GLP-1R agonists depending on both glucose concentration during and before treatment and/or whether the cells have been previously exposed to these drugs. In conclusion, the findings described in this manuscript will be useful in determining in vitro how pancreatic beta cells respond to T2D drug treatments in healthy, diabetic, and recovered states.

## 1. Introduction

Type 2 diabetes (T2D), which is a multifactorial metabolic disease that is largely characterized by impaired insulin secretion and action, has become a global pandemic in recent decades [1,2,3,4]. Despite the availability of various treatments, T2D post-diagnosis complications such as cardiovascular disease (CVD) remain prevalent, and finding long-term stable treatment regimes for patients can be challenging [1,2,3,4,5]. Therefore, novel therapeutic strategies, which alleviate the pathology associated with T2D more effectively than current treatments, are highly desirable. The glucagon-like peptide (GLP)-1 hormone, which is secreted into the circulation by intestinal L-cells in response to food intake, has been shown to augment glucose-stimulated insulin secretion (GSIS) from islet beta cells [1,3]. GLP-1 acts by binding to its receptor (GLP-1R), which is a member of the class B G-protein-coupled receptor (GPCR) family and is expressed abundantly in islet beta cells and to a lesser extent in other tissues [1,3,6]. GLP-1 has a short half-life due to rapid proteolytic degradation by dipeptidyl peptidase (DPP)-IV. Therefore, GLP-1R agonists, such as liraglutide and exenatide, have been developed for use in T2D treatment [3,7]. Liraglutide is a derivative of GLP-1, which has been modified to be resistant to breakdown by DPP-IV. Exenatide is a derivative of exendin-4, which is present in the saliva of the Gila monster lizard, which shares ~53% amino acid identity with GLP-1 and lacks the DPP-IV proteolytic site [1,5]. It has become clear that GLP-1 and its analogues used in T2D therapy mediate multiple systemic physiological beneficial effects in addition to their ability to induce the incretin effect [8,9,10]. Numerous studies have reported beneficial/disease-alleviating effects of GLP-1R agonists on multiple organs, implying that GLP-1 analogues could provide effective treatment strategies in the future for a range of diseases such as dementia, CVD, and kidney disease, both in diabetic and non-diabetic individuals [1,5,6,9,10,11]. Therefore, GLP-1-based therapies are a promising treatment option for the multifactorial disease T2D, given their potential to alleviate the systemic pathology associated with this disease via direct and indirect mechanisms [8,9,10]. In recent years, liraglutide has been approved in the USA to treat obesity, given its ability to induce weight loss in both diabetic and non-diabetic individuals to a greater extent than other drug options and its superior safety [8].

GLP-1 and its mimetics, such as liraglutide and exenatide, exert their actions by binding to the orthosteric binding site of GLP-1R, which results in the activation of both the Gαs and Gαq pathways [1,7,12,13,14]. The activation of the Gαs subunit of heterotrimeric G-protein raises intracellular cAMP levels via adenylyl cyclase (AC) activation, whereas the activation of the Gαq subunit increases intracellular calcium (_i_Ca^2+^) levels and extracellular signal-regulated kinase (ERK) phosphorylation, which promotes GLP-1R internalization [14]. The internalization of GLP-1R is associated not only with the recycling and/or degradation of the receptor but also with raising the cAMP levels [1,7,14]. The agonist-mediated receptor internalization also depletes the agonist levels by causing an intracellular breakdown of the bound ligand [15]. The raised cAMP is necessary for GSIS by increasing the mobility of intracellular insulin secretory granules, which enhances exocytosis [16]. To overcome difficulties associated with the long-term administration of liraglutide and exenatide as injectable drugs for T2D treatment, the orally active small molecule agonists of the GLP-1R such as compound **2** (6,7-dichloro-2-methylsulfonyl-3-*N*-*tert*-butylaminoquinoxaline) and compound **B** (4-(3-(benzyloxy)phenyl)-2-(ethylsulfinyl)-6-(trifluoromethyl)-pyramidine [BETP]) have been developed. They are called allosteric agonists, since their binding site on GLP-1R is spatially and functionally distinct from that of the GLP-1R orthosteric agonists [13]. In contrast to the orthosteric agonists, the allosteric agonists only activate the Gαs pathway [1,7,13,14].

Interestingly, the allosteric agonists have also been shown to act as ago-allosteric modulators of GLP-1R by enhancing GLP-1 binding to the GLP-1R and acting in an additive manner to increase GSIS [17,18]. Compound **B** has been shown to raise insulin secretion to near-normal levels in human islets isolated from a donor with T2D [18] and raise intracellular cAMP levels similar to that of GLP-1 in GLP-1R-expressing HEK293 cells [13]. The allosteric agonists may prolong insulin secretion for a greater duration than orthosteric agonists, as they do not induce GLP-1R internalization [3,7,13,14]. Currently, the importance of the activation of the Gαq pathway downstream of GLP-1R in the orthosteric agonists’ potentiation of GSIS is unclear [1,3,7,13]. Since the Gαq pathway functions by mediating GLP-1R internalization, the main function of this pathway could be to prevent continual and physiologically undesirable orthosteric agonist action. Both GSIS and glucose-stimulated intracellular cAMP (GSICP) are readouts for GLP-1R activation. Therefore, the objective of this study was to compare the GSIS and GSICP potentiation ability of allosteric GLP-1R agonists (compounds **2** and **B**) with that of the orthosteric GLP-1R agonists (liraglutide and exenatide) in healthy, diabetic, and recovered conditions. The rat pancreatic beta cell line INS-1E exhibits GSIS and is responsive to glucose within the physiological range; therefore, it is widely used as a model cell line in vitro for diabetic research [19,20]. Given the limitations and difficulties with using primary pancreatic islet beta cells from patients, such as the availability of samples, variability between samples, and difficulties with culturing the cells, it was, therefore, necessary to use established immortalised cell lines that can be cultured over a long period without changes in the phenotype [19]. Although several pancreatic beta cell lines are currently available, only a few of them (such as the rat islet beta cell line INS-1E and the human pancreatic beta cell line EndoC-βH1) exhibit GSIS and are responsive to glucose within the physiological range [19,21,22]. Since EndoC-βH1 cells are relatively difficult to culture, INS-1E rat pancreatic beta cells were used in this study to analyze GSIS and GSICP under healthy, diabetic, and recovery conditions. Multiple studies have demonstrated that high glucose exposure induces pathology in insulin-producing cells and negatively impacts insulin secretion [23,24,25,26,27,28,29]. Therefore, in this study, ‘diabetic’ cells were produced via chronic exposure of INS-1E cells to glucotoxic conditions. This model of producing ‘diabetic’ cells has clinical relevance. Given that the majority of T2D patients are overweight and that excessive nutrient consumption promotes disease pathogenesis [9], it is reasonable to assume that their islet beta cells experience high glucose exposure during and after the manifestation of this disease.

Initially, the minimum dosage of the orthosteric or allosteric GLP-1R agonist required for maximal augmentation of GSIS and GSICP was determined after 1 h of exposure. All the subsequent experiments then used these concentrations for each agonist during the treatments. Next, the time-dependent effects of the orthosteric and allosteric agonists were analyzed for both GSIS and GSICP in the INS-1E cells. Then, it was tested whether combinations of the GLP-1R orthosteric and allosteric agonists produced any enhanced effects on GSIS and GSICP. Furthermore, how the GLP-1R orthosteric and allosteric agonist pretreatments may have altered GSIS and GSICP upon re-exposure to the agonist treatments was determined. Since raised intracellular cAMP is predominantly responsible for orthosteric agonist action [1,3,7,13,14,16], we also determined what proportion of the GLP-1R agonists’ action was mediated by the elevation of cAMP [14,30,31,32,33]. Finally, to determine the sustainability of these agonists for T2D treatment, the effects of these drugs on intracellular insulin contents were determined after continual treatment for several weeks with GLP-1R agonists. Comparing the results between the healthy, diabetic, and recovered INS-1E cells facilitated the determination of how these agonists may differentially affect healthy individuals, pre-diagnosis T2D patients, and post-diagnosis patients who are on a stable treatment regime, respectively [2,4].

## 2. Results

### 2.1. The GLP-1R Agonists Augment GSIS and GSICP in Healthy, Diabetic, and Recovered INS-1E in a Dose-Dependent Manner

**Figure 1:**
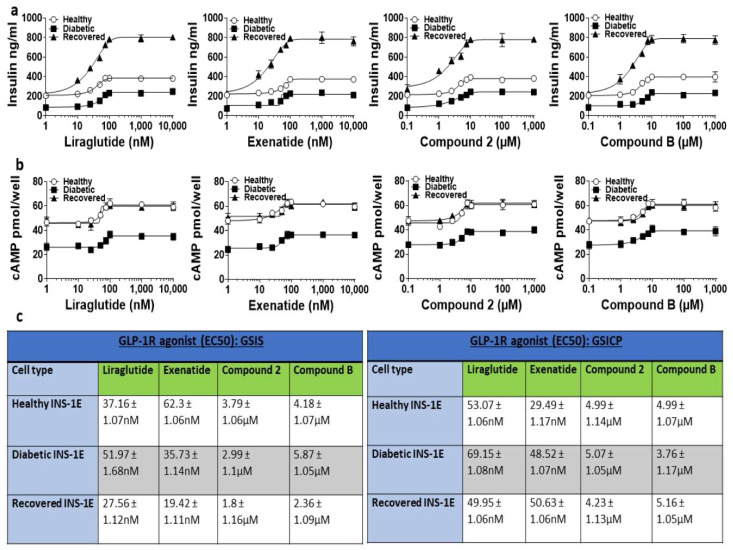
Determining the dose-dependent effects of the GLP-1R agonists on the GSIS and GSICP of healthy, diabetic, and recovered INS-1E cells. INS-1E cells were stimulated with different doses of the GLP-1R agonists (liraglutide, exenatide, compound **2**, and compound **B**) for 1 h in the presence of 15 mM of glucose. Then, they were used for analysing insulin secretion (GSIS (**a**)) and intracellular cAMP contents (GSICP (**b**)). Data are mean ± SEM, *n* = 3. (**c**) The EC50 of GLP1R agonists for insulin secretion and intracellular cAMP contents in healthy, diabetic, and recovered INS-1E cells.

Firstly, the dose-dependent effects of GLP-1R orthosteric agonists (liraglutide and exenatide) and allosteric agonists (compounds **2** and **B**) on the viability of the healthy, diabetic, and recovered rat pancreatic beta cell line (INS-1E) in the presence of 15 mM glucose was determined (Figure S1). The viability of all the cell types exposed to the GLP-1R agonists for 4 h was comparable to that of the cells not exposed to the GLP-1R agonists, which suggests that exposure to GLP-1R agonists is not toxic to these cells. Since GLP-1R agonists are known to augment GSIS and GSICP, the dose-dependent effects and half-maximal effective concentrations (EC50) of the orthosteric and allosteric agonists of GLP-1R on GSIS and GSICP in healthy, diabetic, and recovered INS-1E cells were determined (Figure 1). The EC50 of all the GLP-1 agonists for GSIS and GSICP did not drastically change in the diabetic or recovered conditions. This analysis revealed that the optimal concentration was 100 nM for the orthosteric agonists and 10 μM for the allosteric agonists. The GLP-1R agonists were then used at these concentrations in all further experiments.

### 2.2. Analysing GLP-1R Agonists’ Time-Dependent Augmentation of GSIS and GSICP in Healthy, Diabetic, and Recovered INS-1E Cells

**Figure 2:**
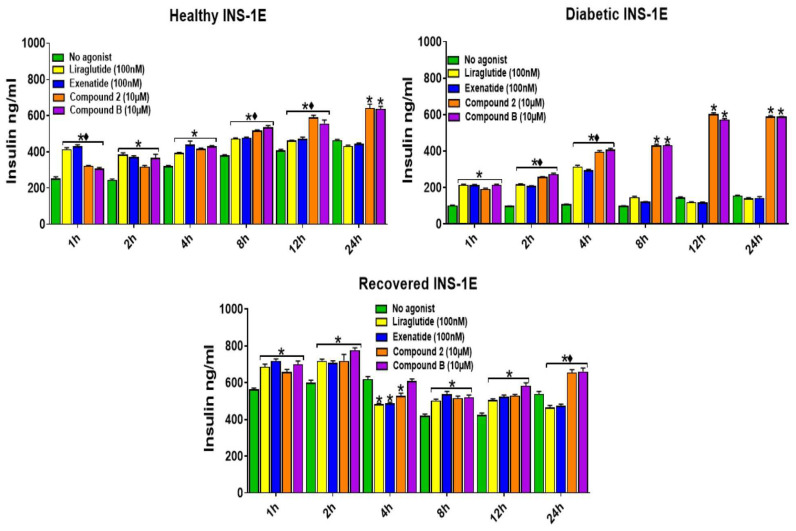
The time-dependent effects of the GLP-1R agonists on GSIS in healthy, diabetic, and recovered INS-1E cells exposed to 15 mM glucose. Healthy, diabetic, and recovered INS-1E cells were stimulated without (no agonist) or with GLP-1R agonists, liraglutide (100 nM), exenatide (100 nM), compound **2** (10 μM), or compound **B** (10 μM), for 1–24 h in the presence of 15 mM glucose. Data are mean ± SEM, *n* = 6. Where both orthosteric and/or allosteric agonists significantly augment/decrease insulin secretion is indicated by * (*p* ≤ 0.05) at specified time points. The black diamonds indicate that the boost/decrease in GSIS by any orthosteric agonist was significantly different * (*p* ≤ 0.05) from the boost induced by any allosteric agonist at that time point. No symbol on the bars indicates non-significance (*p* > 0.05) in comparison to the no agonist control at that time point.

**Figure 3:**
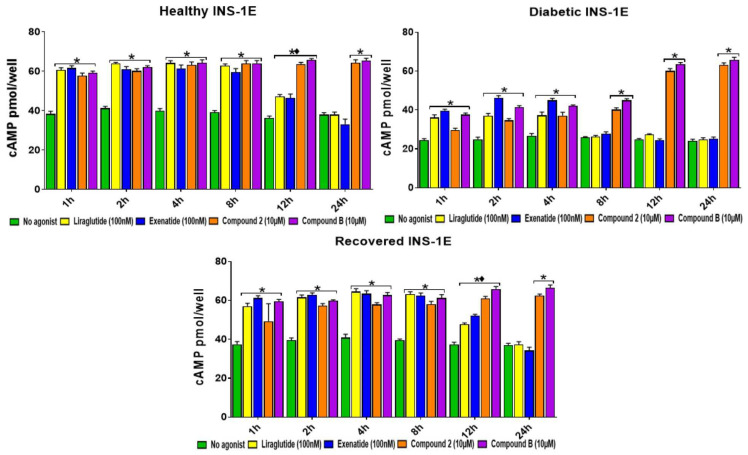
The time-dependent effects of the GLP-1R agonists on GSICP in healthy, diabetic, and recovered INS-1E cells exposed to 15 mM glucose. Healthy, diabetic, and recovered INS-1E cells were stimulated without (no agonist) or with GLP-1R agonists, liraglutide (100 nM), exenatide (100 nM), compound **2** (10 μM), or compound **B** (10 μM), for 1–24 h in the presence of 15 mM glucose. Data are mean ± SEM, *n* = 3. Where both orthosteric and/or allosteric agonists significantly augmented GSICP is indicated by * (*p* ≤ 0.05) at specified time points. The black diamonds indicate that any boost in GSIS induced by any orthosteric agonist was significantly different * (*p* ≤ 0.05) from the boost induced by any allosteric agonist at that time point.

To analyze the time-dependent effects of the orthosteric and allosteric GLP-1R agonists on the GSIS (Figure 2) and GSICP (Figure 3) of INS-1E cells under the healthy, diabetic, and recovered conditions, the cells were incubated with these agonists at the lowest concentration that generated the maximal response for 0–24 h in the presence of 15 mM glucose. In the healthy cells, GSIS was boosted by the orthosteric agonist treatments for 12 h and the allosteric agonist treatments for up to 24 h. In the diabetic cells, the orthosteric agonist treatments for 4 h and the allosteric agonist treatments for 24 h significantly enhanced GSIS. In the recovered INS-1E cells, the orthosteric agonist treatments for 12 h and the allosteric agonist treatments for 24 h significantly boosted GSIS, except for the 4 h treatment, where neither the orthosteric nor allosteric agonists boosted GSIS. Also, the basal insulin secretion by the recovered INE-1E cells was high when compared to that secreted by healthy cells. Interestingly, the treatment of orthosteric agonists for 24 h significantly dampened GSIS in the recovered INS-1E cells. Surprisingly, the greatest enhancement in GSIS via allosteric agonist treatment for 24 h (up to a 5-fold increase over glucose-only treated cells) was observed in the diabetic INS-1E cells. The GSICP was significantly (*p* > 0.05) enhanced by all GLP-1R agonists in a time-dependent manner in all the cell types (Figure 3). The orthosteric and allosteric agonists enhanced GSICP in the healthy and recovered cells up to 12 h and 24 h, respectively, with the allosteric agonists conferring a significantly stronger boost at 12 h. However, in the diabetic cells, the stimulation by orthosteric and allosteric agonists boosted GSICP for 4 h and 24 h, respectively. In all the cell types, the treatment with allosteric agonists for 24 h strongly enhanced GSICP and, in comparison, the orthosteric agonist boost was completely absent. The GLP-1R agonists did not mediate any boost in GSIS at 2.5 mM glucose exposure up to 4 h (Figure S2), demonstrating that the insulinotropic actions of these agonists are dependent on high glucose. However, the agonists were able to boost cAMP production at 2.5 mM glucose, but not to the same extent as what was observed for the 15 mM glucose exposure.

**Figure 4:**
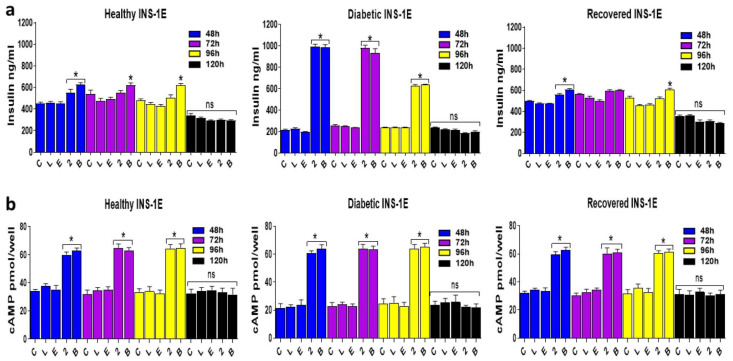
Determining GLP-1R agonists’ augmentation of GSIS and GSICP in healthy, diabetic, and recovered INS-1E cells for 48–120 h. Healthy, diabetic, and recovered INS-1E cells were stimulated without (control [**C**]) or with GLP-1R agonists, liraglutide ([**L**] 100 nM), exenatide ([**E**] 100 nM), compound **2** ([2] 10 μM), or compound **B** ([**B**] 10 μM), for 48–120 h in the presence of 15 mM glucose. The samples were analyzed using insulin (**a**) and cAMP (**b**) ELISAs. Data are mean ± SEM, *n* = 3. At each time point, significantly different values in comparison to the appropriate no-agonist control sample are indicated by the * (*p* ≤ 0.05). Non-significance (*p* > 0.05) between all the samples at any time point is indicated by ns.

Unlike the orthosteric GLP-1R agonists, the allosteric agonists did not induce GLP-1R internalization [13,14]; therefore, their treatment did not alter GLP-1R cell surface expression. We assessed whether there was any correlation between the allosteric GLP-1R agonists prolonging insulin secretion and GLP-1R cell surface expression in INS-1E cells under healthy, diabetic, and recovered conditions (Figure S3). As expected, the treatment with the orthosteric agonists, but not the allosteric agonists, for up to 12 h significantly reduced GLP-1R cell surface expression. Interestingly, treatment with the same agonists for 24 h resulted in the cell surface levels of GLP-1R returning to what was observed for the glucose-only treated cells. The GLP-1R mRNA (Figure S4) and protein cell surface expression (Figure S3) were higher in the diabetic cells. To gain further insight into the longevity of the action of allosteric GLP-1R agonists, the effects of GLP-1R agonists for a longer period (48 h to 120 h) on GSIS (Figure 4a) and GSICP (Figure 4b) were analyzed in the presence of 15 mM glucose in INS-1E cells under the healthy, diabetic, and recovered conditions. At 48 h, 72 h, and 96 h of stimulation, both compounds **2** and **B** slightly increased GSIS in the healthy and recovered INS-1E cells and significantly boosted GSIS in the diabetic INS-1E cells. However, there was no indication of any orthosteric agonist boosting GSIS after 48 h, 72 h, and 96 h of stimulation in any cell type. At 120 h of stimulation, no agonist was able to boost GSIS (*p* > 0.05) in the INS-1E cells. After 48 h, 72 h, and 96 h of stimulation, orthosteric augmentation of GSICP was absent, whereas both allosteric agonists boosted GSICP. At 120 h of stimulation, neither allosteric agonists nor orthosteric agonists augmented GSICP.

### 2.3. The Time-Dependent Effects of GLP-1R Agonists in Combination with GSIS and GSICP in Healthy, Diabetic, and Recovered INS-1E Cells

**Figure 5:**
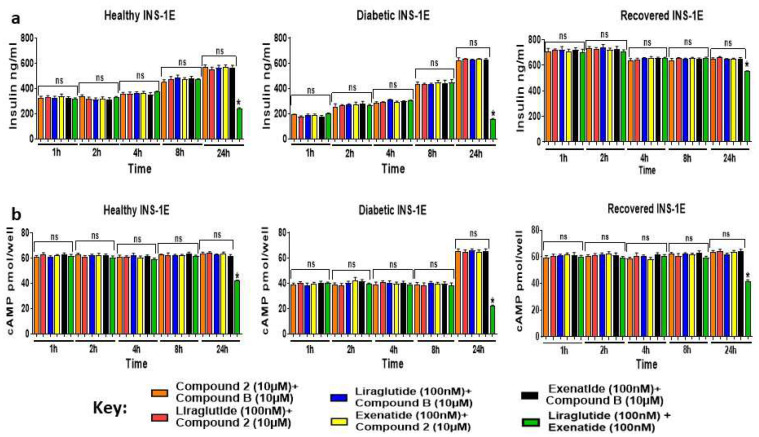
Assessing the combining effects of two GLP-1R agonists on GSIS and GSICP in healthy, diabetic, and recovered INS-1E cells. Healthy, diabetic, and recovered INS-1E cells were stimulated either simultaneously with one orthosteric (liraglutide [100 nM] or exenatide [100 nM]) and one allosteric (compound **2** [10 μM] or compound **B** [10 μM]) GLP-1R agonist or two orthosteric (liraglutide [100 nM] and exenatide [100 nM]) or allosteric (compound **2** [10 μM] and compound **B** [10 μM]) GLP-1R agonists for 1–24 h in the presence of 15 mM glucose. Samples were analyzed using insulin (**a**) and cAMP (**b**) ELISAs. Data are presented as mean ± SEM, *n* = 3. At each time point, statistical non-significance between different condition samples is indicated by ns (*p* > 0.05), and statistical significance is indicated by * (*p* ≤ 0.05).

To determine whether there were any synergistic effects of combining orthosteric and allosteric GLP-1R agonists on GSIS and GSICP, healthy, diabetic, and recovered INS-1E cells were treated with the GLP-1R agonists’ different combinations for 1–24 h in the presence of 15 mM glucose (Figure 5a). There was no indication of any synergistic effects of combining both types of GLP-1R agonists on GSIS, as the levels of GSIS augmented by the agonists in combination were similar (*p* > 0.05) to that observed for the individual agonist treatments at any time point (Figure 2). The GLP-1R allosteric and orthosteric agonists either in combinations (Figure 5b) or alone (*p* > 0.05) (Figure 3) augmented GSICP similarly in the cells under all three conditions when they were stimulated for 1–24 h. However, the effects of the orthosteric GLP-1R agonist combinations on GSIS and GSICP diminished when the cells were treated with them for 24 h. The data here suggest that no synergetic effects were produced by the combination of the GLP-1R orthosteric and allosteric agonist stimulations on GSIS or GSICP in healthy, diabetic, or recovered INS-1E cells.

### 2.4. Analysing GLP-1R Agonist Augmentation of GSIS and GSICP in Healthy, Diabetic, and Recovered INS-1E Cells Stimulated a Second Time with the Same Agonists

**Figure 6:**
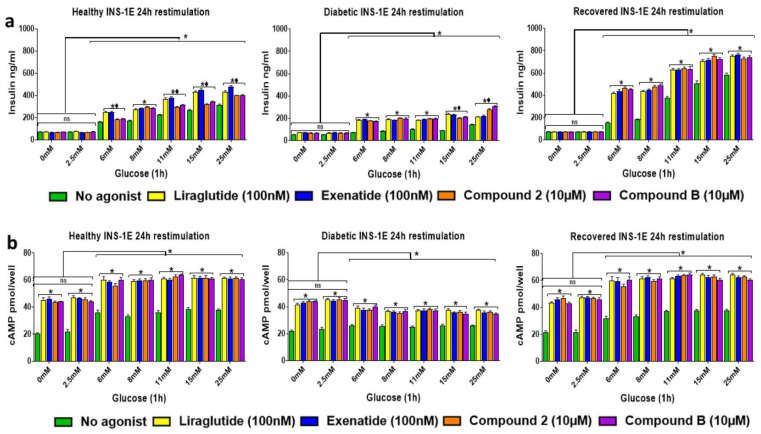
Assessing insulin secretion in INS-1E cells exposed a second time to the same GLP-1R agonists. Healthy, diabetic, and recovered INS-1E cells were stimulated twice (with a 24 h gap between them) with GLP-1R agonists, liraglutide (100 nM), exenatide (100 nM), compound **2** (10 μM), or compound **B** (10 μM) in the presence of 0–25 mM glucose. Samples were then obtained after 1 h for analysis using insulin (**a**) and cAMP (**b**) ELISAs. Data are mean ± SEM, *n* = 3. Non-significance (*p* > 0.05) between samples is indicated by ns. Significance between all samples from 6–25 mM glucose exposure vs. corresponding samples at 0 and 2.5 mM glucose exposure is indicated by * (*p* ≤ 0.05). At each different glucose concentration, significantly higher values in comparison to the appropriate glucose control sample are indicated by * (*p* ≤ 0.05). The black diamonds indicate that both orthosteric and allosteric agonist values were significantly different * (*p* ≤ 0.05) from each other at each glucose concentration.

Since T2D patients need to take GLP-1R agonist-based medication regularly, we analyzed whether restimulating for 1 h of healthy, diabetic, and recovered INS-1E cells that had been treated 24 h earlier with the same agonists produced any altered responses (GSIS (Figure 6a) and GSICP (Figure 6b)). The GLP-1R agonists’ augmentation of GSIS and GSICP in healthy and recovered INS-1E cells during restimulation was comparable to that yielded during stimulation for the first time (see Figure 2 and Figure 3). In the diabetic cells, the GLP-1R agonists also induced GSIS to a similar extent to what was seen in the cells during primary stimulation, but this was not the case for GSICP during restimulation.

### 2.5. Analyzing the Effects of 1 h Preincubation with 0 or 8 mM Glucose on GLP-1R Augmentation of GSIS and GSICP in All Cell Types

**Figure 7:**
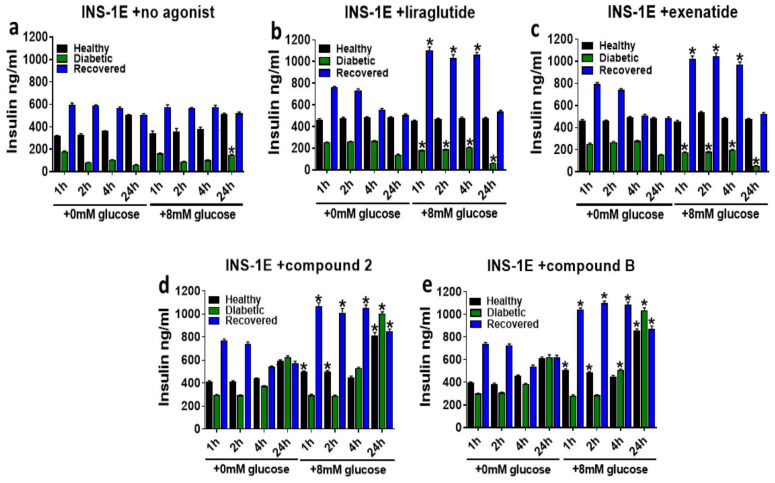
Assessing the effects of 1 h preincubation with 0 or 8 mM glucose on GLP-1R agonists’ augmentation of GSIS in healthy, diabetic, and recovered INS-1E cells. INS-1E cells of each type (healthy, diabetic, and recovered) were preincubated in KRH buffer with either 0 mM or 8 mM glucose for 1 h before treating them with 15 mM glucose without (**a**) or with the GLP-1R agonist (100 nM liraglutide (**b**), 100 nM exenatide (**c**), 10 μM compound **2** (**d**), or 10 μM compound **2** (**e**)) for 1–24 h. Data are mean ± SEM, *n* = 3. At each time point, significantly different values at 8 mM compared to 0 mM glucose prestimulation within each cell type (healthy, diabetic, and recovered) are indicated by the * (*p* ≤ 0.05), which was obtained via an unpaired *t*-test analysis.

To determine whether differential responses are produced by preincubating healthy, diabetic, and recovered INS-1E cells with different concentrations of glucose before the GLP-1R agonist treatments, the cells were either incubated with KRH buffer containing either 0 or 8 mM glucose for 1 h before treating the cells with GLP-1R agonist in the presence of 15 mM glucose (Figure 7). Firstly, we assessed any alterations in GSIS induced by preincubations with 0 or 8 mM glucose only (Figure 7a). Interestingly, GSIS was comparable (*p* > 0.05) in the diabetic cells between 0 and 8 mM glucose prestimulated glucose-only treated cells at all time points except 24 h (Figure 7a), where GSIS was significantly enhanced in the 8 mM glucose preincubated diabetic cells. However, in both the healthy and recovered INS-1E cells, GSIS in the cells preincubated with 8 mM glucose was comparable (*p* > 0.05) at all time points. In the diabetic cells treated with allosteric agonists (Figure 7d,e), GSIS was either comparable (1, 2, and 4 h) or significantly (*p* ≤ 0.05) enhanced (24 h) in the cells preincubated with 8 mM glucose. In contrast to what was observed for the allosteric agonists, the orthosteric agonists impaired GSIS significantly (*p* ≤ 0.05) in the diabetic INS-1E cells preincubated with 8 mM glucose (Figure 7b,c). In the healthy INS-1E cells treated with orthosteric agonists (Figure 7b,c), GSIS was comparable between the cells stimulated with 0 and 8 mM glucose. The allosteric agonists augmented GSIS to a greater extent (*p* ≤ 0.05) in healthy INS-1E cells preincubated with 8 mM glucose at all time points apart from 4 h of stimulation (Figure 7d,e). In the recovered INS-1E cells, preincubation with 8 mM glucose significantly (*p* ≤ 0.05) enhanced GSIS at all time points of the stimulation with the allosteric and orthosteric agonist treatments, although the effects of the orthosteric agonists were lost after 24 h of treatment (Figure 7b–e).

**Figure 8:**
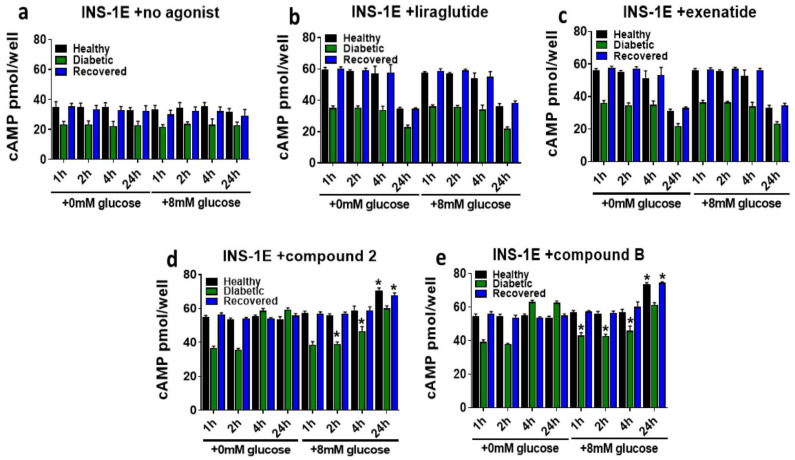
Assessing the effects of 1 h of preincubation with 0 or 8 mM glucose on GLP-1R agonist augmentation of GSICP in healthy, diabetic, and recovered INS-1E cells. INS-1E cells of each type (healthy, diabetic, and recovered) were preincubated in KRH buffer with either 0 mM (**a**) or 8 mM glucose for 1 h before treatment with 15 mM glucose without or with the GLP-1R agonists (100 nM liraglutide (**b**), 100 nM exenatide (**c**), 10 μM compound **2** (**d**), or 10 μM compound **2** (**e**)) for 1–24 h. Data are mean ± SEM, *n* = 3. At each time point, significantly different values at 8 compared to 0 mM glucose prestimulation within each cell type (healthy, diabetic, and recovered) are indicated by * (*p* ≤ 0.05), which was obtained using an unpaired *t*-test analysis.

Next, we assessed any alterations in time-dependent GSICP induced by the GLP-1R agonist treatments in the healthy, diabetic, and recovered INS-1E cells preincubated with 0 or 8 mM of glucose (Figure 8). The GSICP values of the 0 and 8 mM glucose-preincubated healthy, diabetic, and recovered INS-1E cells were comparable (Figure 8a). This was also the case for the orthosteric agonist treatments (Figure 8b,c). The allosteric agonist treatment results were similar between cells preincubated with 0 and 8 mM glucose after 1 and 2 h, apart from compound **B**, which boosted GSCIP to a greater extent in the diabetic cells preincubated with 8 mM glucose in some instances (Figure 8d,e). GSCIP is significantly reduced as a result of allosteric agonist treatment in diabetic cells preincubated with 8 mM glucose after 4 h. After 24 h of stimulation with any GLP-1R agonist, the GSCIP was similar between the 0 and 8 mM preincubations in the diabetic cells, but in the healthy and recovered cells, the GSCIP was significantly enhanced in the 8 mM preincubated cells treated with the allosteric agonists.

### 2.6. Comparison of IBMX and Forskolin Potentiation of GSIS and GSICP with That by GLP-1R Agonists in Healthy, Diabetic, and Recovered INS-1E Cells

**Figure 9:**
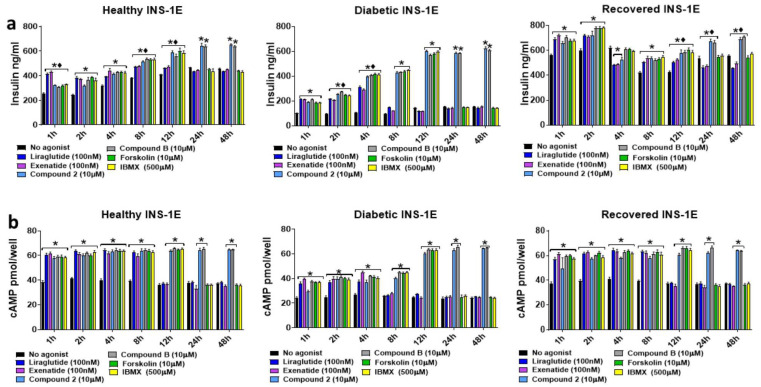
Assessing the time-dependent effects of GLP-1R agonists and cAMP-elevating chemicals (IBMX and forskolin) on GSIS and GSICP in healthy, diabetic, and recovered INS-1E cells. Healthy, diabetic, and recovered INS-1E cells were stimulated with GLP-1R agonists, liraglutide (100 nM), exenatide (100 nM), compound **2** (10 μM), compound **B** (10 μM), IBMX (10 μM), or forskolin (0.5 mM) for 1–48 h in the presence of 15 mM glucose. Samples were analyzed using insulin (**a**) and cAMP (**b**) ELISAs. Data expressed as mean ± SEM, *n* = 3. At each time point, significantly different values in comparison to the no agonist control sample are indicated by * (*p* ≤ 0.05). The black diamonds indicate that any boost in GSIS induced by the orthosteric agonists was significantly different * (*p* ≤ 0.05) from the boost induced by the allosteric agonists at that time point. No symbol on the bars indicates non-significance (*p* > 0.05) in comparison to the no agonist control at that time point.

Given that IBMX and forskolin augment intracellular cAMP levels, and that raised cAMP is associated with GSIS [14,16,30,31,32,33], we compared the orthosteric and allosteric GLP-1R agonists’ time-dependent augmentation of GSIS and GSICP with that of these chemicals in the INS-1E cells in all three conditions (Figure 9). As observed before (see Figure 2), the basal insulin secretion by the recovered cells was relatively high. Like the allosteric agonists, forskolin and IBMX raised intracellular cAMP levels and augmented GSIS to similar levels (*p* > 0.05) up to 12 h. Augmentation of GSIS and GSICP by IBMX and forskolin was lost after 12 h, whereas they continued for 48 h in the allosteric agonist-treated samples. The orthosteric agonists showed superiority over the allosteric agonists, IBMX and forskolin in GSIS augmentation at 1 h in only healthy INS-1E cells. The augmentation of GSICP by orthosteric agonists was comparable (*p* > 0.05) to that of the allosteric agonists or IBMX or forskolin until 8 h, and thereafter, the orthosteric agonist action was absent in all conditions.

### 2.7. Analysis of Intracellular Insulin Contents in Healthy, Diabetic, and Recovered INS-1E Cells Treated for One Day to Four Weeks with the GLP-1R Agonists

**Figure 10:**
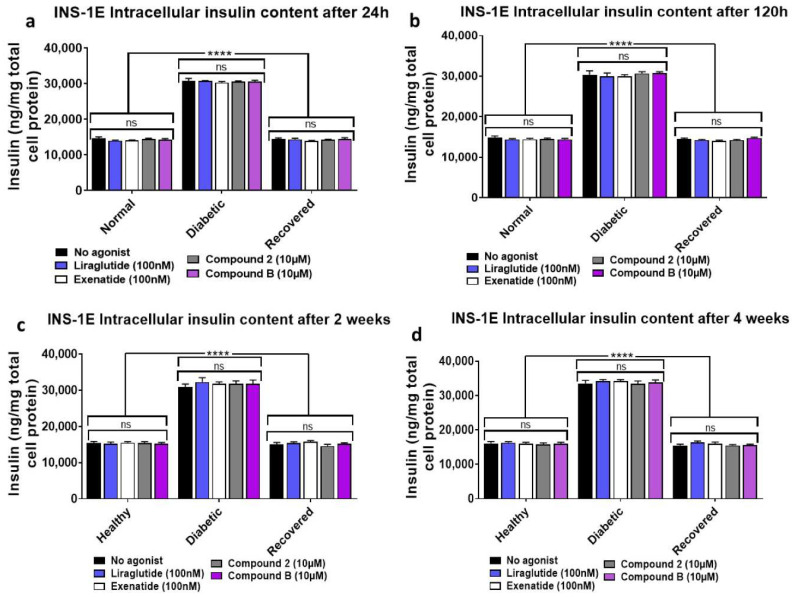
Analysis of intracellular insulin contents in healthy, diabetic, and recovered INS-1E cells either 24 or 120 h after treatment with the GLP-1R agonists or 2 and 4 weeks of continual treatments of these cells with the agonists. Cells of each type (healthy, diabetic, and recovered) from each cell line were lysed with HCl treatment after stimulation for 24 (**a**) or 120 h (**b**) in KRH in the presence of 11 mM glucose with or without GLP-1R agonists. During culturing, cells of each type (healthy, diabetic, and recovered) were exposed to repeated treatments with the GLP-1R agonists for 2 (**c**) or 4 weeks (**d**), either daily treatments for orthosteric agonists or supplementation of medium with the desired concentration of allosteric agonists every 3 days. After the treatment, the cells were then lysed with HCl, and samples were analyzed using an insulin ELISA. Data are mean ± SEM, *n* = 3. All diabetic samples were found to be significantly elevated (**** *p* ≤ 0.0001) in comparison to the healthy and recovered samples. Non-significance (*p* > 0.05) between samples within each cell type is indicated by ns. Statistical analysis was carried out using a two-way ANOVA with Tukey’s post hoc test.

To determine whether treatment with the orthosteric and allosteric GLP-1R agonists for a longer period depletes intracellular insulin levels differently in different types of cells, the intracellular insulin contents of healthy, diabetic, and recovered INS-1E cells were analyzed after they were treated with the GLP-1R agonists for either 24 and 120 h or continual treatments with these agonists over a two- and four-week period (Figure 10). These data show that neither orthosteric nor allosteric GLP-1R agonists alter intracellular insulin contents even after continual treatments with these agonists over four weeks, suggesting that these agonists do not deplete intracellular insulin levels even after long exposure of the cells to these agonists. Diabetic cells have significantly elevated intracellular insulin contents in comparison to what was observed in healthy and recovered cells.

## 3. Discussion

In this study, the ability of GLP-1R orthosteric and allosteric agonists to augment GSIS and GSICP in INS-1E pancreatic beta cells under healthy, diabetic, and recovered states was investigated to gain insight into their therapeutic applications for T2D treatment. It has been suggested that INS-1 cell death is enhanced when they are cultured in high glucose for >48 h [23,27,29]. However, Shi et al. showed that INS-1 cell death is increased only when they are cultured intermittently but not continuously in a high glucose-containing medium [34]. We also found that INS-1E cells survive at 40 mM glucose for up to 4 weeks (Figure S5); therefore, this concentration of glucose was used to generate ‘diabetic’ cells in this study. Given that diabetic cells had significantly lower insulin and cAMP readouts in the majority of settings in this study, it is reasonable to assume that diabetic pathology is induced in these cells. Since the recovered cells displayed similar or increased insulin secretion compared to the healthy cells in response to the treatments in this study, it was appropriate to label these cells as ‘recovered’. Comparable to our observations here, other studies have used chronic hyperglycemia to generate diabetic human pancreatic beta cells or mice [35,36]. Further, the beta cell changes found in diabetic mice were prevented through the restoration of euglycaemia with insulin, indicating that beta cell pathology can be reversed by exposing them to euglycaemic conditions [35]. Both orthosteric and allosteric agonists of GLP-1R augmented GSIS and GSICP in a dose-dependent manner at 15 mM glucose after 1 h of treatment in healthy, diabetic, and recovered INS-1E cells (Figure 1). Subsequent experiments found that GSIS and GSICP were significantly augmented/decreased in a time-dependent manner by the GLP-1R orthosteric and allosteric agonists for up to 12 h in all three conditions (Figure 2 and Figure 3). The allosteric agonist augmentation of GSIS and GSICP was still present in all conditions at 24 h, whilst the orthosteric agonist action was absent or reduced, demonstrating the action of allosteric agonists for a longer duration. The two types of agonists boosted GSIS similarly in INS-1E diabetic and recovered cells at 1 h, but the orthosteric agonist treatments conferred superiority in GSIS augmentation in the healthy cells.

The GLP-1R orthosteric agonists activate both the Gαs and Gαq pathways, whereas allosteric agonists activate only Gαs activity [13,14]. However, orthosteric agonists only showed higher GSIS-augmenting activity than that of the allosteric agonists at 1 h of treatment with healthy cells (Figure 2). These observations imply that GLP-1R internalization further enhances insulin secretion at 1 h in addition to the Gαs activity in healthy cells. Surprisingly, by far, the greatest enhancement in GSIS via allosteric agonist treatment was observed at 24 h in the diabetic cells, which implies that chronic activation of Gαs can greatly boost insulin secretion in diabetic conditions (Figure 2). GSICP was significantly enhanced by all GLP-1R agonists in all cell types, with healthy and recovered cells having similar cAMP levels at all time points, indicating the requirement for additional factors for GLP-1R agonists augmented GSIS, as the recovered cells exhibited greater insulin secretion than the healthy cells (Figure 3). Interestingly, the GSICP levels were lower in the diabetic cells exposed to the GLP-1R agonists than what was observed in the healthy and recovered cells, implying that defective cAMP production may play a role in T2D pathology. In agreement with these observations, previous studies have reported defects in cAMP generation in INS-1E cells exposed to high glucose and GLP-1 [37] and its contribution to impaired insulin secretion in neonatal rats with diabetes (induced by streptozotocin treatment, which destroys the majority of pancreatic beta cells) [38].

In this study, we confirmed that orthosteric and allosteric agonists mediate their effects via different mechanisms, as the orthosteric agonist treatment resulted in significantly reduced GLP-1R cell surface expression, presumably due to the induction of GLP-1R internalization, whereas the allosteric agonist treatment did not affect GLP-1R cell surface expression (Figure S3). However, the increased insulin secretion up to 12 h of the orthosteric agonist treatments (Figure 2) suggests that the Gαq activity can further augment insulin secretion. The loss of orthosteric agonist action after 12 h is likely due to agonist degradation through GLP-1R internalization, resulting in orthosteric agonist levels becoming depleted. Consistent with this, the internalization of GLP-1R was lost in the cells treated with the orthosteric agonists for 24 h (Figure S3). The increase in mRNA (Figure S4) and cell surface expression (Figure S3) of GLP-1R in the ‘diabetic’ cells implies that the activity of this receptor could be important for the survival of these cells exposed to high glucose. To further investigate the relatively longer action of allosteric GLP-1R agonists, we analyzed these agonists’ augmentation of GSIS and GSICP for a longer period (2–5 days) (Figure 4). At 48–96 h but not after 96 h, only allosteric agonists augmented GSIS and GSICP. We speculate that the longevity of the allosteric agonists’ action is due to their inability to induce GLP-1R internalization, which is required for their degradation [1]. Therefore, in contrast to orthosteric agonists, allosteric agonists likely enable prolonged Gαs activation, which in turn promotes continual cAMP production and sustained augmentation of GSIS and GSICP.

The augmentation of GSIS and GSICP by individual GLP-1R agonist treatments was not altered when they were used in combination, suggesting that no synergistic effects are produced by combining allosteric and orthosteric agonists at the optimal concentration (Figure 5). The allosteric agonists have been shown to act as ago-allosteric modulators of GLP-1R by enhancing GLP-1 binding to the GLP-1R, which results in increased GSIS and GSICP induced by GLP-1 [13,17,18]. Although we were not able to determine whether they enhance orthosteric agonist binding to GLP-1R in this study, we showed that allosteric agonists do not enhance orthosteric agonist-mediated GSIS or GSICP (Figure 5). This could be due to the use of orthosteric agonists at the optimal concentration, which produces a maximal response; hence, there is no possibility of further increase in the response by allosteric agonists. This suggests future investigation into comparing the effects of orthosteric and allosteric GLP-1R agonists, either alone or together at sub-optimal concentrations, on insulin secretion. When INS-1E cells were re-stimulated with the GLP-1R agonist, all the agonists significantly augmented both GSIS and GSICP in the healthy and recovered cells, and only GSIS was augmented in the diabetic cells (Figure 6).

Prestimulating the healthy, diabetic, and recovered INS-1E cells with 8 mM glucose, as opposed to 0 mM, yielded different results with the GLP-1R agonist treatments (Figure 7 and Figure 8). It seems that these cells responded differently to both glucose and agonist treatments depending on what conditions they had been previously exposed to. Therefore, these observations suggest that patient islet beta cell response to T2D treatments will likely be influenced by blood glucose levels at the time of drug administration. IBMX and forskolin augment intracellular cAMP levels, and raised cAMP levels are known to be associated with increased insulin secretion in response to high glucose [14,16,30,31,32,33]. Therefore, in this study, the time-dependent augmentation of GSIS and GSICP by the GLP-1R agonists and the cAMP-elevating agents IBMX and forskolin was compared to determine what proportion of the augmentation by GLP-1R agonist action is mediated by elevation of cAMP (Figure 9). The similarity in augmentation of GSIS and GSICP between the GLP-1R agonists and the cAMP-elevating chemicals suggests that all of these predominantly/exclusively mediate their effects on GSIS via augmentation of cAMP through the Gαs signalling. This is an important observation, as it has been previously unclear what signalling pathways, either Gαs or Gαq, are most important for full orthosteric GLP-1R-mediated augmentation of GSIS and how the allosteric agonists’ action, which activates only the Gαs pathway, compares with this [1,3,7,13,14]. The reduced/absent effect of the orthosteric agonists and cAMP-elevating chemicals at the later time points is likely due to the agonist degradation associated with GLP-1R internalization [14] and inferior stability, respectively, in comparison to the allosteric agonists. The superiority of orthosteric agonist action over allosteric/IBMX/forskolin is only demonstrated on GSIS at 1 h in healthy INS-1E cells, in agreement with previous observations in this study (Figure 2), which implies that the Gαq signalling could be important in this scenario.

This study also suggests that the GLP-1R agonist treatment does not deplete intracellular insulin content or reduce viability in healthy, diabetic, or recovered INS-1E cells (Figure 10 and Figure S1). This indicates that intracellular insulin turnover and synthesis are ongoing during the treatments, which is needed to replace the insulin that is secreted for the cell to be able to meet future demand. Therefore, the results of this study indicate that both orthosteric and allosteric agonists are sustainable and safe treatment options for T2D. However, to properly investigate the sustainability of allosteric agonists, understanding how these drugs alter intracellular insulin over a much greater period than what was investigated in this study with continual treatments is necessary. Interestingly, the impaired GSIS observed in the diabetic model seems to be due to defective mechanisms of insulin secretion, given the increased intracellular insulin content in the diabetic cells. However, this is in contrast to what other studies have reported, where high glucose exposure reduces the expression of insulin [25,28,29]. Given that the purpose of this study was to establish the potential of allosteric agonists for T2D treatment, the results obtained for the ‘diabetic’ cells in this study have high clinical relevance [2]. To our knowledge, this is the first study that has focused on analysing ‘recovered’ INS-1E cells’ responses to GLP-1R agonists. This also has clinical relevance, as post-diagnosis hyperglycaemia is typically alleviated in patients once they are placed on stable treatment regimes, which then results in their islet beta cells being exposed to nutrient conditions in circulation that mirror healthy individuals to some extent [4]. Additionally, the results obtained for the ‘recovered’ cells treated with GLP-1R agonists in this study may enable insight into how islet beta cell behavior changes in individuals in T2D remission as a result of bariatric surgery or low-calorie diets [39,40]. To further understand how GLP-1R agonists augment GSIS and GSCIP in INS-1E cells, future studies should repeat the experiments of this study at a range of glucose concentrations in addition to 15 mM, which was used in this study. Additionally, determining how the optimal concentration of GLP-1R agonists needed to induce maximal GSIS and GSCIP varies at different time points after 1 h, which was not done in this study, will be useful to gain further insight into how these agonists mediate their effects in a time-dependent manner. Given that being overweight and consuming excessive nutrients are associated with T2D pathogenesis [9], the generation of ‘diabetic’ cells in this study via chronically high glucose exposure seemed a reasonable in vitro model to generate ‘diabetic’ cells. However, in vivo studies with higher clinical relevance using similar methodologies are needed to further support any clinical implications from this study.

## 4. Materials and Methods

### 4.1. Materials

Liraglutide and exenatide working concentrations were obtained by diluting Victoza^®^ (Novo Nordisk UK, London, UK) 6 mg/mL (1.6 mM) and Byetta^®^ (Astra Zeneca UK, Cambridge, UK) 250 μg/mL (60 μM) solution from pre-filled clinically used injectable pens, respectively. All other chemicals, including compounds **2** (DMB) and **B** (BETP) and consumables were obtained from Merck unless otherwise stated. INS-1E cells were provided by Pierre Maechler (University of Geneva, Switzerland) [20].

### 4.2. INS-1E Cell Culturing

Rat INS-1E cells were cultured in RPMI-1640 containing 11 mM glucose and supplemented with 10% foetal bovine serum (FBS), penicillin (100 U/mL), streptomycin (0.1 mg/mL), glutamine (2 mM), sodium pyruvate (1 mM), HEPES (10 mM [pH 7.4]), and β-mercaptoethanol (50 μM) (full serum medium [FSM]) by incubating them in a humid incubator at 37 °C and 5% CO_2_ [20]. The cells were maintained in sterile 10 cm^2^ tissue culture Petri dishes (Greiner Bio-one, Stonehouse, UK) by changing the FSM every 3–4 days and sub-culturing when they reached 100% confluency. Diabetic and recovered INS-1E cells were generated using a previously described method with some changes [41]. To produce ‘diabetic’ INS-1E cells, when healthy INS-1E cells reached >80% confluency, they were maintained in FSM containing high glucose (40 mM) for 4 weeks by changing the medium with high glucose every 3–4 days. To produce ‘recovered’ cells, the healthy cells were cultured initially in FSM containing 40 mM glucose (diabetic condition) for 4 weeks and then in FSM containing 11 mM glucose for 4 weeks. For all the following assays, INS-1E cells (100,000 cells/well) were plated in a 96-well plate in FSM and incubated for 24 h in a humid incubator at 37 °C and 5% CO_2_ for the cells to attach and reach >80% confluency. Unless otherwise specified, all incubations of the cells in the following assays were carried out in a humid incubator at 37 °C and 5% CO_2_, and all of the ELISAs were carried out at room temperature (RT).

### 4.3. Insulin ELISA

The insulin secreted by INS-1E cells during the treatments was measured using an insulin ELISA. INS-1E cells (100,000 cells/well) were plated in a 96-well plate in FSM. After 24 h, the cells were pre-incubated in Krebs–Ringer–HEPES (KRH) buffer (129 mM NaCl, 4.8 mM KCl, 1.2 mM MgSO_4_, 1.2 mM KH_2_PO_4_, 2.5 mM CaCl_2_, 5 mM NaHCO_3_, 10 mM HEPES [pH 7.4]) containing 0.5% fish gelatin for 1 h, washed twice with KRH, and then incubated for 1 h in KRH to ensure that residual insulin was removed. The cells were then incubated in the KRH buffer containing the specified concentration of glucose without or with the specified concentration of GLP-1R agonist(s) for the desired time. After cessation of the cell treatments, the media were clarified via centrifugation at 22,000× *g* for 5 min and then diluted 1 in 25 in dilution buffer (0.5% fish gelatin made in PBST [PBS with 0.05% tween-20]) and used for the insulin ELISA. The sample preparation for measuring the intracellular insulin contents of the INS-1E cells via the insulin ELISA was as follows. After cessation of the treatments, the cells were lysed in 150 μL of 0.1 M HCl. The cell lysates were centrifuged at 22,000× *g* for 5 min, then the supernatant was diluted 1 in 1000 in dilution buffer to use as the samples for the insulin ELISA.

For the ELISA, the wells of 96-well plate inserts were coated with 50 μL of 3 μg/mL captured antibody (Novus Biologicals, Abingdon, UK) diluted in PBS for 2 h at 37 °C and then washed four times with PBST. The antibody-coated wells were then blocked for 1 h with 150 μL of blocking buffer (5% fish gelatin made in PBST). Samples of 50 μL of samples were added to the capture antibody-coated wells in duplicate and incubated overnight at 4 °C. The wells were then washed five times with PBST and incubated with 50 μL of HRP-conjugated detection antibody (Abcam; diluted 1/10,000 in dilution buffer) for 2 h. The wells were washed again five times with PBST and incubated with 50 μL of Ultra TMB substrate (Thermo Fisher Scientific, Loughborough, UK) for 5 min. The reaction was stopped by adding an equal volume of 2M sulfuric acid, then its optical density was read at 450 nm (OD_450_) using a plate reader. Using known insulin standards (Abcam, Cambridge, UK), the insulin concentrations of samples were determined.

### 4.4. cAMP ELISA

The intracellular cAMP contents of the INS-1E cells during the treatments was measured using a cAMP ELISA. INS-1E cells (100,000 cells/well) were plated in a 96-well plate in FSM. After 24 h, the cells were pre-incubated with KRH buffer containing 0.5% fish gelatin for 1 h, washed twice with KRH and then incubated for 1 h with KRH to reduce basal cAMP levels. The cells were incubated in the KRH buffer containing various concentrations of glucose without or with different concentrations of GLP-1R agonists for the desired time. The cells were then lysed in 150 μL of 0.1M HCl by incubating at RT for 20 min, and then the cell lysates were centrifuged at 16,000× *g* for 10 min. A total of 12 μL of the supernatant was neutralized with 6 μL of 0.2M NaOH, diluted to 120 μL with TBS containing 0.05% Tween-20 (TBST), and used in the cAMP ELISA.

For the ELISA, an anti-rabbit antibody (Genscript, Oxford, UK) diluted to 10 μg/mL in 100 mM NaHCO_3_ buffer (pH 9.6) was used to coat the wells (50 μL per well) of a 96-well plate overnight at 4 °C. The coated wells were washed twice with TBST and blocked with blocking buffer (TBST containing 0.5% fatty acid-free BSA) for 1 h. Samples of 50 μL were then added individually in duplicate to the antibody-coated wells. After 1 h of incubation, 25 μL of anti-cAMP rabbit polyclonal antibody (Genscript) diluted 1/10,000 in blocking buffer was added to each well, and the samples were incubated for 1 h. A total of 25 μL of HRP-conjugated cAMP (Genscript) was then added (diluted 1/10,000 in blocking buffer) to the wells, and the samples were incubated for 1 h in the dark. The wells were washed five times with TBST and then 50 μL of Ultra TMB was added to each well. The reaction was stopped after 5 min with 50 μL of 2M sulfuric acid, then its optical density was read at 450 nm (OD_450_) using a plate reader. Using known cAMP standards, the cAMP contents of the samples were determined.

### 4.5. Cell Counting Kit-8 (CCK-8) Assay

The CCK-8 assay (Dojindo Laboratories, Insight Biotechnology, Wembley, UK) was used to determine the viability of the cells after the treatments. INS-1E cells (100,000 cells/well) were plated in a 96-well plate in FSM and incubated for 24 h in a humid incubator at 37 °C and 5% CO_2_ for the cells to attach and reach >80% confluency. Before commencing the treatments, the cells were washed twice with KRH buffer and incubated for 1 h in KRH-containing 2.5 mM glucose. The cells were then incubated in the KRH buffer containing the various treatments for 4 h. CCK-8 reagent was added at a 1/10 dilution to each well, and the cells were incubated in a humid incubator at 37 °C and 5% CO_2_ for 4 h with this solution. Finally, the solution OD_450_ was read using a plate reader.

### 4.6. Statistical Analysis

Data were analysed using the GraphPad Prism program. All the data are presented as means ± standard error of the mean (SEM) of three or more independent experiments. One-way or two-way analyses of variance (ANOVA) were used for all statistical analyses in this study with Tukey’s post hoc tests to compare the data between multiple groups. A *p*-value of >0.05 was considered statistically not significant (ns). Statistical significance is shown as * *p* < 0.05, ** *p* < 0.01, *** *p* < 0.001, and **** *p* < 0.0001.

## 5. Conclusions

Overall, this study suggests that the GLP-1R allosteric agonists have a much greater duration of action than the orthosteric agonists, which gives them a clinical advantage over current GLP-1R-based therapies. This study also indicates that the GLP-1R agonists’ treatment does not deplete intracellular insulin at the dosage that mediates maximum effect, meaning they should be a sustainable and safe treatment option for T2D. This study also provides evidence that both orthosteric and allosteric GLP-1R agonists predominantly augment GSIS through the elevation of intracellular cAMP. Furthermore, we demonstrated how cells may respond variably to the GLP-1R agonists depending on the glucose concentration during and before treatment and/or whether cells have been previously exposed to these drugs. Additionally, we demonstrated how cells differentially respond to treatments depending on whether they are in healthy, diabetic, or recovered states. The highly variable and dynamic behavior of INS-1E cells needs to be considered when determining the effectiveness of new therapies for T2D using this cell line. The outcomes of this study may influence future studies that aim to determine how INS-1E cells respond to drug treatments for diseases such as T2D.

## Data Availability

Data is contained within the article and Supplementary Materials.

## Supplementary Materials

The following supporting information can be downloaded at: https://www.mdpi.com/article/10.3390/ijms25126331/s1. Ref. [42] is cited in Supplementary Materials file.
