# Supplementary material for: Comparative Analysis of Orthosteric and Allosteric GLP-1R Agonists’ Effects on Insulin Secretion from Healthy, Diabetic, and Recovered INS-1E Pancreatic Beta Cells
Source: Int J Mol Sci. 2024 Jun 7;25(12):6331. doi: 10.3390/ijms25126331 (PMC11203424; doi:10.3390/ijms25126331)
Supplement: Supplementary file 1 [file ijms-25-06331-s001.zip › ijms-2963600-supplementary.pdf]

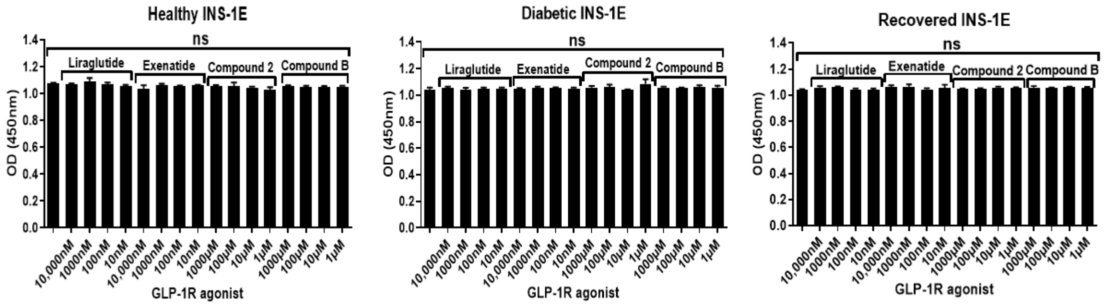

Figure S1: Determining the dose-dependent effects of the GLP-1R agonists on the viability of healthy, diabetic, and recovered INS-1E. Healthy, diabetic, and recovered INS-1E cells were stimulated with different doses of the GLP-1R agonists (liraglutide, exenatide, compound 2, and compound B) in the presence of 15 mM glucose. After 4 h incubation, the viability of the INS-1E cells was assessed using the CCK-8 assay. Data are mean ±SEM, n = 3 (ns, non-significant).

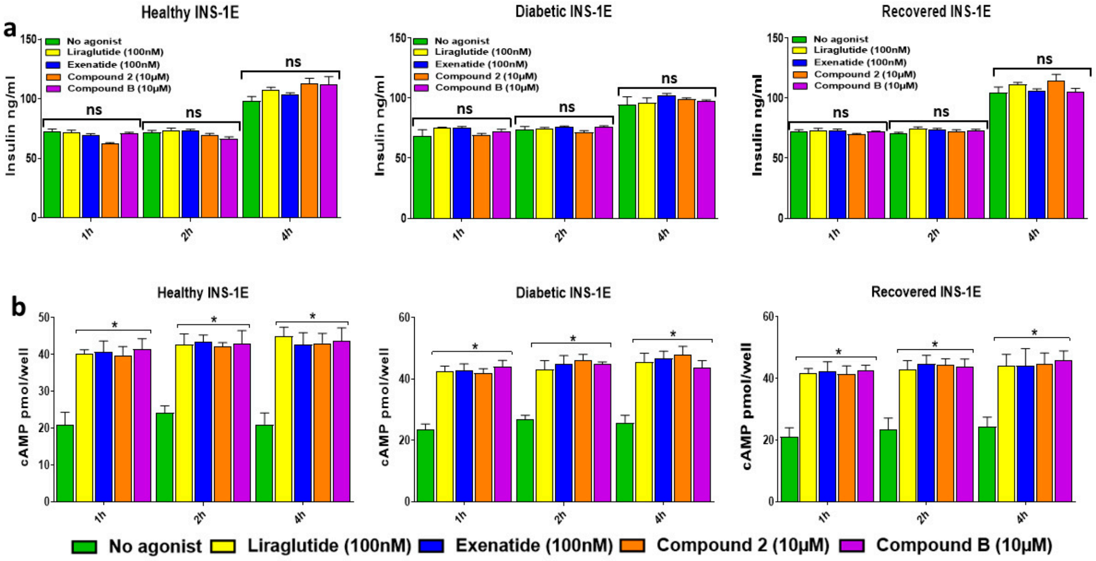

Figure S2: The time-dependent effects of the GLP-1R agonists on GSIS and GSICP in healthy, diabetic, and recovered INS-1E cells exposed to 2.5 mM glucose. Healthy, diabetic, and recovered INS-1E cells were stimulated without (no agonist) or with GLP-1R agonists, liraglutide (100 nM), exenatide (100 nM), compound 2 (10 μM), or compound B (10 μM), for 1–4 h in the presence of 2.5 mM glucose. The samples were analyzed using insulin (a) and cAMP (b) ELISAs. Data are mean ± SEM, n = 3. At each time point, statistical non-significance between different condition samples is indicated by ns ($p > 0.05$) or statistical significance by * ($p \leq 0.05$).

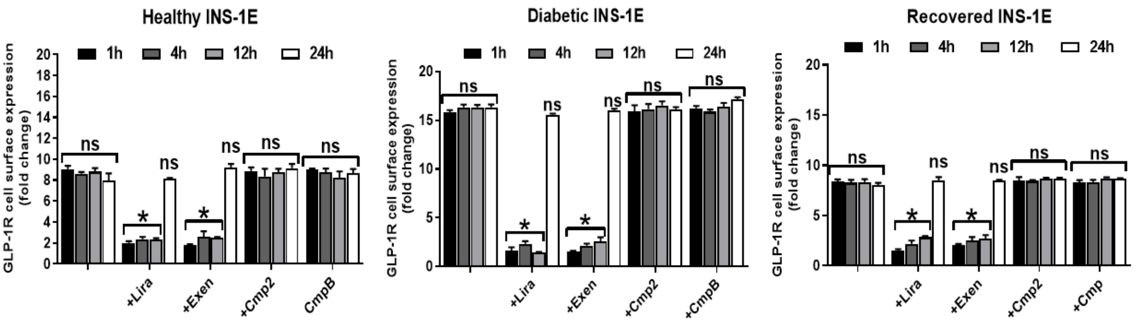

Figure S3: Determining the time-dependent effects of the orthosteric and allosteric GLP-1R agonists on GLP-1R internalization in healthy, diabetic, and recovered INS-1E cells. Healthy, diabetic, and recovered INS-1E cells were stimulated with the GLP-1R agonists (orthosteric [100 nM liraglutide (Lira) or exenatide (Exen)] or allosteric (10 µM compound 2 [Cmp2] or compound B [CmpB]) for 1–24 h in the presence of 15 mM glucose. After the stimulation, the cells were stained with a GLP-1R antibody [42], without permeabilising them, and cell surface staining was analyzed using flow cytometry. Data are shown as fold-change in GLP-1R cell surface expression levels calculated by normalizing to cell surface staining by isotype control. Data are mean ± SEM, n = 3. Significantly different values in comparison to untreated cells at each time point are indicated by the * ($p \leq 0.05$). NS indicates non-significance ($p > 0.05$) between no-agonist control and agonist-treated samples.

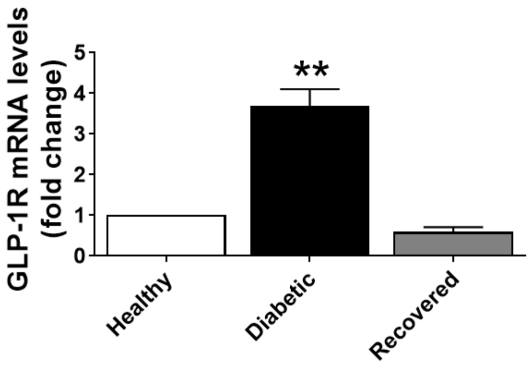

Figure S4: Analysis of GLP-1R mRNA expression in healthy, diabetic, and recovered INS-1E cells. Total RNA isolated from normal, diabetic, and recovered INS-1E using Triazol was converted into cDNA using reverse transcriptase. The cDNAs were subjected to RT-PCR using the GLP-1R primers (forward: 5′-GTCATCTTTGCCTTTGTGATGGACG-3′; reverse: 5′-CTGGACCTCATTGTTGACAAAGCAG-3′). Data are mean ± SEM, n = 3. Diabetic INS-1E cells had significantly higher expression of GLP-1R mRNA than normal and recovery INS-1E cells of the same cell line, indicated by ** ($p \leq 0.01$).

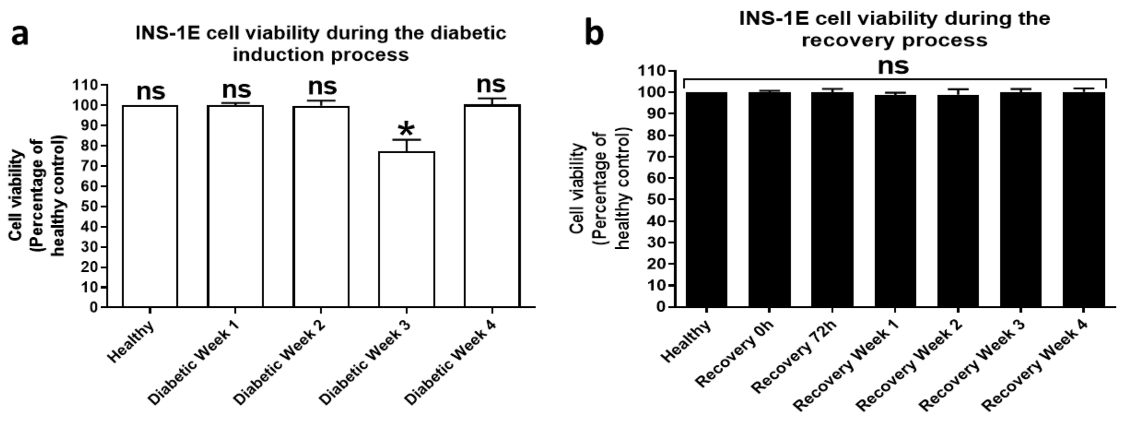

Figure S5: Determining the viability of the rat pancreatic beta cell line (INS-1E) during the diabetic and recovery processes. The cells were exposed to 40 mM glucose for the indicated periods to make them diabetic (a), and the diabetic cells were exposed to 11 mM glucose for an indicated period during the recovery process (b). Cell viability was assessed using the CCK-8 assay. Cells were incubated with CCK-8 reagent for 4 h at either 11 mM (healthy and recovery) or 40 mM (diabetic) glucose during the assay. Data are mean ± SEM, n = 3. Significantly different values in comparison to healthy cells are indicated by * ($p \leq 0.05$). Non-significance ($p > 0.05$) between samples is indicated by ns.
